# A randomized trial of comparing video telecare education vs. in-person education on dietary regimen compliance in patients with type 2 diabetes mellitus: a support for clinical telehealth Providers

**DOI:** 10.1186/s12902-022-01032-4

**Published:** 2022-05-02

**Authors:** Shahram Molavynejad, Mojtaba Miladinia, Mina Jahangiri

**Affiliations:** 1 Diabetes Research Center, Health Research Institute, Ahvaz Jundishapur University of Medical Sciences, Ahvaz, Iran; 2 Student Research Committee, Ahvaz Jundishapur University of Medical Sciences, Ahvaz, Iran; 3 Department of Biostatistics, Faculty of Medical Sciences, Tarbiat Modares University, Tehran, Iran

**Keywords:** Tele-education, Telehealth, Self-care, Telecare, Telemedicine

## Abstract

**Background:**

Compliance to dietary recommendations by patients is the most difficult part of diabetes management. The nature of any educational method is to increase patients’ awareness. But the question is, what is the effect of each method and for this purpose a comparative method should be considered. Therefore, this study was conducted to compare the effects of in-person education versus video tele-education on dietary regimen compliance in patients with T2DM.

**Methods:**

In this trial, 378 patients with type 2 diabetes mellitus (T2DM) were random allocated into video tele-education, in-person education and control groups. The patients’ weight and biochemical parameters were measured before educational programs and three-month later.

**Results:**

The mean changes of patients’ weight, glycemic parameters, and Lipid profiles decreased more in the two educational groups than the control group in a three-month period. There were no significant differences in the all study variables between the in-person and video education groups in post interventions except Total Cholesterol (TC). The pre- and post-intervention changes in the weight, TC, hemoglobin A1c, Triglyceride, and Very Low-density Lipoprotein Cholesterol were significant in both in-person group and video group. None of the educational programs had a significant impact on the Fasting blood sugar, Low-Density Lipoprotein Cholesterol, and High-Density Lipoprotein Cholesterol.

**Discussion:**

Video tele-education was just as effective as in-person educational method on dietary regimen compliance among patients with T2DM in a three-month period. Therefore, it is recommended to use video tele-education in combination with or as an alternative to the in-person education method. This study provides support for diabetes educator.

**Trial registration:**

This investigation was registered in the Iranian Registry of Clinical Trials Center (IRCT20150302021307N4).

**Supplementary information:**

The online version contains supplementary material available at 10.1186/s12902-022-01032-4.

## Introduction

Diabetes Mellitus is a serious chronic illness that requires daily self-care decisions made by patient [1, 2]. The Standards of American Diabetes Association Introduces diabetes self-management education (DSME) as an inseparable aspect of the care for diabetic patients [3]. Diet modification and compliance to dietary recommendations by patients is the most difficult part of diabetes management and are suggested as the first steps for diabetes’ control [4]. Despite the positive effects of dietary recommendations and lifestyle modifications in patients with diabetes, the number of patients who follow such recommendations is declining [5], which is currently a major issue for the diabetes care and education specialists [6, 7]. Patients’ insufficient knowledge of Type 2 Diabetes Mellitus (T2DM) and failure in maintaining a healthy diet are the some of the explanations why patients do not comply with dietary recommendations [8]. Chronic elevate blood glucose levels can result in several complications and reduction in the patient’s quality of life [9, 10]. Previous interventions have shown that appropriate education and training can result in higher rates of compliance to dietary guidelines [11, 12].

In-person/face-to-face education is among the most common methods of patient education. However, face-to-face education requires the presence of the patient and nurses at the same location, and spending large amounts of time to deliver the instruction, has become increasingly impossible in over-crowded healthcare centers in Iran. Given the current advancements in technology, the use of innovative methods such as using tele-educational technology for the training patients with chronic diseases is required. Today, telecare is becoming part of nursing care. Tele-education impacts the self-care process by focusing on the role of patients and increasing their involvement, independence, self-confidence, and feeling of security [13]. Video for distance learning is one of the telehealth-related education for training patients [14, 15]. Video tele-education technology can be used as telehealth education in nursing. The application of video telecare education to provide patients with information about basic health concepts is suggested [16]. The advantages of the use of video technology are the storage of a large amount of information, reducing anxiety during training [17, 18], help to compliance to self-care recommendations, ability to watch multiple times, and simple and low-cost applications [19].

Few studies have investigated the effect of video education on the awareness, patients’ satisfaction [20–23], and the compliance to therapeutic regimens and self-care activities [23–26] in patients with diabetes. The findings of these studies showed that video education is beneficial on the awareness of patients with diabetes, improving their paraclinical parameters and self-care in diabetic foot. The nature of any educational method is to increase patients’ awareness. But the question is, what is the effect of each method and for this purpose a comparative method should be considered. Therefore, this study was conducted to compare the effects of face-to-face education versus video telecare education on dietary regimen compliance in patients with T2DM for supporting the diabetes care and telehealth Providers.

## Methods

### Design and settings

**Fig. 1:**
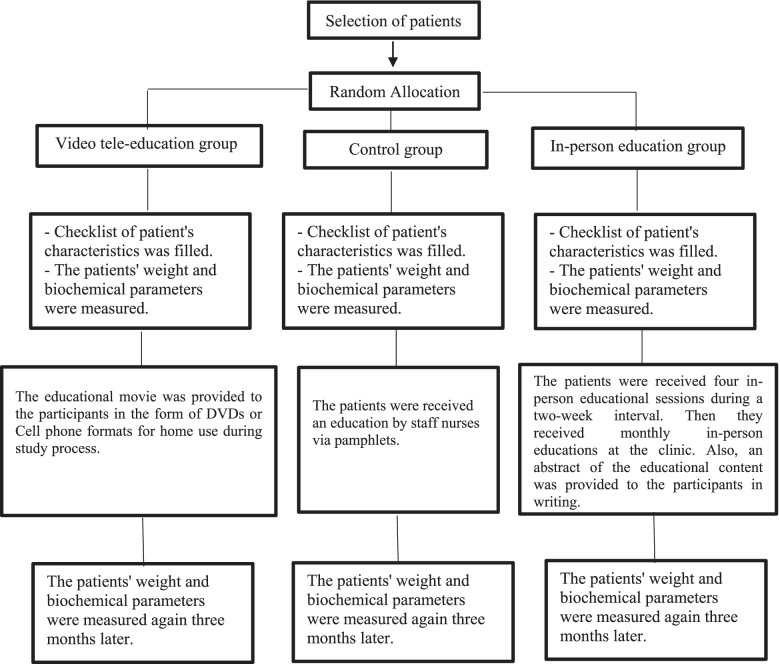
Summary of study protocol

This 3-arm randomized controlled trial was conducted between 2020 and 2021 in a diabetes healthcare clinic affiliated with the Ahvaz Jundishapur University of Medical Sciences (AJUMS) in the southwest of Iran. Figure 1 shows the summary of study protocol.

### Participants and sample size

A sample size of 125.88 in each group, based on Cohen’s d (with 80% power, a 5% level of significance, effect size 0.2 and 15% drop-out), was calculated with PASS software. 378 patients were selected based on the following inclusion criteria: (1) the history of T2DM at least for one year; (2) age above 18 years; (3) having no other chronic diseases; (4) receiving no official education on self-care before or attending other educational program during this study; (5) not being pregnant or under breastfeeding. Also, those patients who were absent in two educational sessions, their health condition was worsening, were no longer interested in participation in the study, hospitalized during the study or changed their therapeutic regimen were excluded from continuing the study process.

**Fig. 2:**
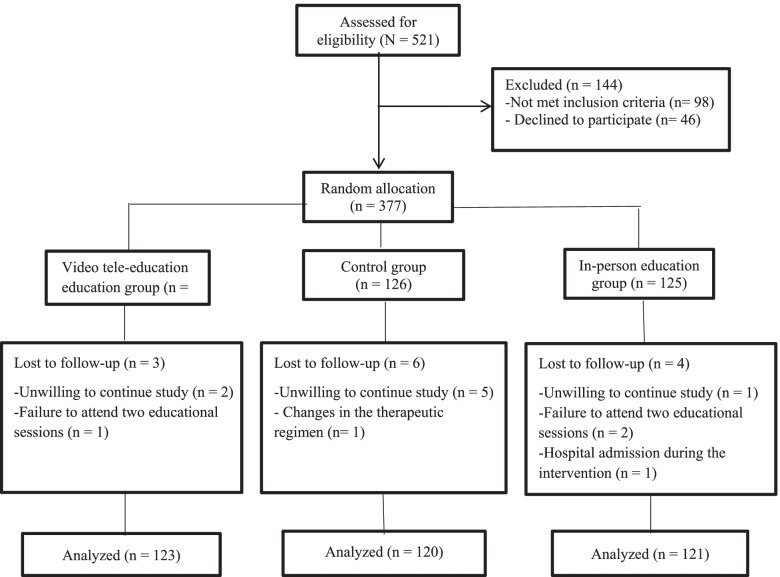
Consort flow diagram

The patients were randomly allocated into either the video education (*n* = 126), control (*n* = 126), and in-person education (*n* = 126) group using a permuted block randomization method. The statistician generated the random allocation sequence and a research assistant enrolled and assigned participants. It was noted that during the study, three, six, and five patients were excluded from the video education, control, and in-person education groups, respectively due to various reasons (Fig. 2).

### Intervention

The educational programs were prepared with the help of an endocrinologist, a nutritionist, and two clinical nurses who were specialists in the provision of care to patients with T2DM. The educational content included the definition of T2DM, its symptoms, signs and complications, the effects of dietary regimen compliance, nutritional balance and dietary recommendations, cooking methods, weight control, periodical check-ups, and blood glucose self-monitoring. The educational programs were delivered in both Farsi and Arabic languages, because the research zone was a city in which both Farsi and Arabic speakers lived. Therefore, two educational programs were developed and implemented through face-to-face education and video telecare education. The educational contents were similar for both the in-person and video education groups. A coordinator nurse was adjusting the schedule for the educational sessions.

- In-person education group: the patients were received oral face-to-face education via group sessions. The participants were divided into several subgroups. Each subgroup received four educational sessions during a two-week interval (two sessions per each week). The educational program was presented to them via lectures in a quiet conference room. At the end of each 45-minute session, there was a discussion opportunity using a question-and-answer format. The face-to-face education was performed by two clinical nurses who were diabetes education specialists. Then they received monthly in-person education at the clinic. Also, an abstract of the educational content was provided to the participants in writing for home use.
- Video telecare education group: the participants were divided into several subgroups. For the first time, the educational movie was displayed in the clinic for all subgroups. After the end of the movie display, there was a discussion opportunity using a question-and-answer format. The total movie length was two hours. The educational movie divided into several episodes was provided to the participants in the form of DVDs or Cell phone formats for home use (distance learning) during study process. During the study, research assistants reminded participants via regular text messages to watch the video education again and contact if they had any questions.
- Control group: the participants were received an education by staff nurses via pamphlets including education about lifestyle management of T2DM.

After the study, patients in the control group received the in-person or video telecare educational programs based on their own preference. Also, the participants in the face-to-face education group were offered the video education.

### Measurements

Before the initiation of the study, every participant completed a demographic questionnaire through a face-to-face interview and checked for completeness by a medical record review. In order to evaluate the effectiveness of educational methods on dietary regimen compliance, changes in patients’ weight and biochemical parameters were measured in a three-month period. To prepare for the trial, before the educational intervention, patients’ weight and biochemical parameters such as glycemic parameters [Fasting Blood Sugar (FBS), Glycated Hemoglobin A1c (HbA1c)], and Lipid profiles [Total Cholesterol (TC), High-Density Lipoprotein Cholesterol (HDL), Low-Density Lipoprotein Cholesterol (LDL), Very Low-Density Lipoprotein Cholesterol (V-LDL) and Triglyceride (TG) levels] were measured. The biochemical parameters and patients’ weight were measured again three months later as post-interventions data. The Lipid profiles and also HbA1c by using the enzymatically method, and the FBS by using the enzymatic photometric test (GOD-PAP) method were measured. Moreover, the patients in the intervention groups were asked to score their satisfaction with the educational program on a scale from 0 to 10.

### Blinding

The participants were unaware of educational programs administered to the other groups. Also, nurses who collected blood samples, laboratory technicians and the statistician were blinded to group assignment.

### Data analysis

The Kolmogorov-Smirnov test showed normality of the quantitative variables. One-way ANOVA was used to compare mean scores between the groups. In addition, the LSD Post-Hoc test was used for multiple comparisons of mean score differences. Paired t-test was employed to compare mean score values between the pre-test and post-test in each group. The waterfall plots in the educational groups were drawn to display each patient’s response in terms of weight changes compared with baseline. The statistical analysis was performed using descriptive and inferential statistics with R statistical software. *P* < 0.05 was considered statistically significant.

## Results

### Demographic and disease-related characteristics

The majority of the participants were employed (43.4%), married (85.2%), lived in an urban environment (86.3%), and had up to high school education (48.6%). Moreover, the majority was able to control their diabetes using oral medications (63.5%). There were no statistically significant differences in baseline characteristics between groups (Table 1).

**Table 1:** The patient's demographic and disease-related characteristics in the baseline

Characteristic	In-person Group (***n*** = 121)	Control Group (***n*** = 120)	Video Group (***n*** = 123)	***P***-value
**Age (Mean**±SD**)**	47.37 ± 7.07	48.31 ± 7.61	45.88 ± 9.09	0.058
**Duration of diabetes (years)**	5.10 ± 5.43	5.43 ± 5.38	6.34 ± 5.12	0.172
**Sex**				
Male	61(50.4%)	65(54.2%)	50(40.7%)	0.093
Female	60(49.6%)	55(45.8%)	73(59.3%)	
**Occupation status**				
Employed	53(43.8%)	60(50.0%)	34(27.6%)	
Unemployed	48(39.7%)	45(37.5%)	65(52.8%)	0.008*
Retired	20(16.5%)	15(12.5%)	24(19.5%)	
**Marital status**				
Married	101(83.5%)	98(81.7%)	111(90.2%)	
Single	12(9.9%)	18(15.0%)	4(3.3%)	0.025*
Divorced/widow	8(6.6%)	4(3.3%)	8(6.5%)	
**Level of education**				
Less than high school	39(32.2%)	42(35.0%)	29(23.6%)	
High school	59(48.8%)	53(44.2%)	65(52.8%)	0.352
Academic education	23(19.0%)	25(20.8%)	29(23.6%)	
**Treatment regime**				
In-person drugs	76(62.8%)	66(55.0%)	89(72.4%)	
Insulin	24(19.8%)	27(22.5%)	16(13.0%)	0.165
In-person drug and insulin	12(9.9%)	16(13.3%)	13(10.6%)	
Diet control	9(7.4%)	11(9.2%)	5(4.1%)	
**Residence**				
Urban	110(90.9%)	105(87.5%)	99(80.5%)	0.054
Rural	11(9.1%)	15(12.5%)	24(19.5%)	

- *SD* (Standard deviation), *n* (Number)

- One-Way ANOVA test and Chi-square test were used.

- * Statistically significant as *P* < 0.05

### Dietary regimen compliance (lipid profiles)

There were no significant differences in the mean levels of Lipid profiles between study groups in the baseline. After a three-month period, a significantly different between three groups were observed for TC and V-LDL indices (Table 2). There were no significant differences in the Lipid profiles between the in-person and video telecare education groups in both pre- and post-intervention (Table 3).

**Table 2:** Between Groups and within Groups comparisons in terms of Lipid profiles, glycemic parameters and weight

Parameters	In-person Group (***n***=121)	Video Group (***n***=123)	Control Group (***n***=120)	***P***-value
**TG**				
Pretest	191.24±90.52	183.54±72.18	188.91±72.62	0.737
Posttest	174.60±58.83	168.02±67.19	187.76±71.14	0.061
***P***_**value**_	0.001*	0.005*	0.085	
**HDL**				
Pretest	43.40±7.50	42.59±7.18	43.72±7.39	0.466
Posttest	44.01±6.67	43.42±6.91	43.82±7.30	0.799
***P***_**value**_	0.290	0.144	0.181	
**LDL**				
Pretest	116.04±33.28	108.59±26.14	113.81±28.19	0.129
Posttest	111.28±38.71	104.86±28.26	113.14±27.66	0.106
***P***_**value**_	0.060	0.130	0.159	
**V-LDL**				
Pretest	38.56±18.32	36.17±15.53	38.05±16.91	0.510
Posttest	31.69±12.90	30.99±13.24	37.10±16.84	0.002*
***P***_**value**_	0.001*	0.001*	0.065	
**Total cholesterol**				
Pretest	196.76±39.60	187.28±36.60	193.80±39.76	0.148
Posttest	190.70±47.99	180.24±34.69	193.23±39.01	0.033*
***P***_**value**_	0.022*	0.026*	0.058	
**FBS**				
Pretest	165.99±64.40	166.65±83.54	162.32±79.57	0.893
Posttest	159.02±59.13	162.67±61.06	161.88±78.07	0.904
***P***_**value**_	0.163	0.528	0.257	
**HbA1c%**				
Pretest	7.62±1.54	7.90±1.88	7.82±1.88	0.453
Posttest	6.78±1.34	6.99±1.59	7.86±1.90	0.001*
***P***_**value**_	0.001*	0.001*	0.060	
**Weight (Kilograms)**				
Pretest	90.12±15.01	93.76±15.28	92.52±16.17	0.178
Posttest	87.74±14.76	91.23±14.77	91.20±16.49	0.129
***P***_**value**_	0.001*	0.001*	0.001*	

- *SD* Standard deviation, *TG* Triglyceride mg/dL, *HDL* High-Density Lipoprotein Cholesterol mg/dL, *LDL* Low-Density Lipoprotein Cholesterol mg/dL, *V-LDL* Very Low-Density Lipoprotein Cholesterol mg/dL, *FBS* Fasting blood Sugar mg/dL, *HbA1c* Glycated Hemoglobin A1c%

- One-Way ANOVA test and Paired t-test were used

- * Statistically significant with a *p* value < 0.05

**Table 3:** The pairwise comparisons of mean differences between Groups

Parameters		In-person Group (***n*** = 121)	Video Group (***n*** = 123)
**TG**	**In-person**	-	P pre (0.447) / P post (0.437)
	**Control**	P pre (0.819) / P post (0.043*)	P pre (0.597) / P post (0.020*)
**HDL**	**In-person**	-	P pre (0.390) / P post (0.512)
	**Control**	P pre (0.736) / P post (0.831)	P pre (0.232) / P post (0.660)
**LDL**	**In-person**	-	P pre (0.051) / P post (0.117)
	**Control**	P pre (0.557) / P post (0.651)	P pre (0.232) / P post (0.660)
**V-LDL**	**In-person**	-	P pre (0.271) / P post (0.704)
	**Control**	P pre (0.815) / P post (0.004*)	P pre (0.388) / P post (0.001*)
**TC**	**In-person**	-	P pre (0.056) / P post (0.047*)
	**Control**	P pre (0.553) / P post (0.631)	P pre (0.189) / P post (0.014*)
**FBS**	**In-person**	-	P pre (0.946) / P post (0.669)
	**Control**	P pre (0.709) / P post (0.740)	P pre (0.658) / P post (0.926)
**HbA1c%**	**In-person**	-	P pre (0.220) / P post (0.327)
	**Control**	P pre (0.393) / P post (0.001*)	P pre (0.713) / P post (0.001*)
**Weight (Kg)**	**In-person**	-	P pre (0.067) / P post (0.077)
	**Control**	P pre (0.230) / P post (0.082)	P pre (0.523) / P post (0.98)

- *P pre P* value of pretest, *P post P* value of posttest, *TG* Triglyceride mg/dL, *HDL* High-Density Lipoprotein Cholesterol mg/dL, *LDL* Low-Density Lipoprotein Cholesterol mg/dL, *V-LDL* Very Low-Density Lipoprotein Cholesterol mg/dL, *TC* Total Cholesterol mg/dL, *FBS* Fasting Blood Sugar mg/dL, *HbA1c* Glycated Hemoglobin A1c%, *Kg* Kilograms

- Post Hoc Multiple Comparisons by LSD was used

- * Statistically significant was set a *p* value < 0.05

In the face-to-face education group showed significant reductions in the mean of TC, TG, and V-LDL after three-month period than the baseline (*p* = 0.022, *p* = 0.001, and *p* = 0.001, respectively). While, in the in-person education group the mean LDL and HDL changes were not significant (*p* = 0.06 and *p* = 0.29, respectively) (Table 2). In the video tele-education group, significant reductions in the mean levels of TC, TG, and V-LDL were observed after three-month period than the baseline (*p* = 0.026, mean changes=-7.03; *p* = 0.005, mean changes=-15.52; *p* = 0.001, mean changes=-5.17). However, the mean LDL and HDL did not significantly change after the video tele-education (*p* = 0.130, mean changes=-3.73 mg/dL; p = 0.144, mean changes = + 0.83 mg/dL, respectively) (Table 2). In contrast, in the control group, mean changes in the Lipid profiles were not significant after a three-month period compared with the baseline (Table 2).

### Dietary regimen compliance (glycemic parameters)

There were no significant differences in the mean levels of glycemic parameters between study groups in the baseline. None of the educational programs had a significant impact on the FBS level (Table 2). In contrast to the FBS, between the study groups, post-intervention difference in HbA1c was significant (Table 2). Reductions in HbA1c were significantly greater in the two educational groups than the control group (Table 3). Moreover, differences in HbA1c were not significant between the in-person and video tele-education groups in both pre-intervention (*p* = 0.220) and post-intervention (*p* = 0.327) (Table 3). The pre- and post-intervention changes in HbA1c were significant in the in-person group (*p* = 0.001, mean changes=-0.84%) and video group (*p* = 0.001, mean changes=-0.91%) (Table 2).

### Dietary regimen compliance (patients’ weight)

**Fig. 3:**
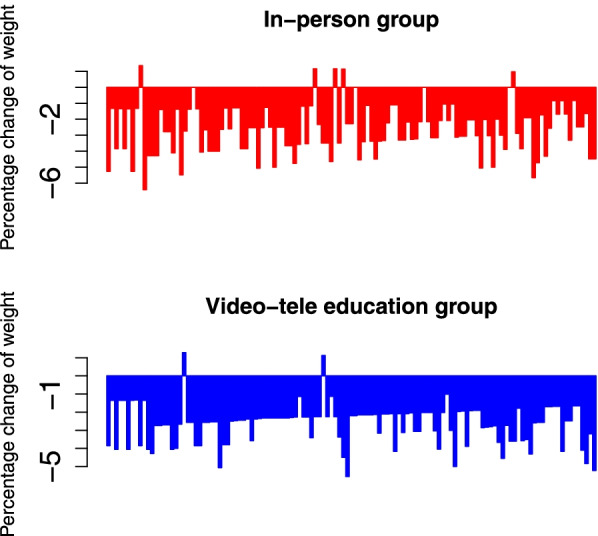
The waterfall plots show each individual patient’s response. The *x*-axes show each participant and the *y*-axes show the percentage of changes in patients’ weight compared with baseline in the educational groups

There were no significant differences in the patients’ weight between the study groups in both pre- and post-intervention (Tables 2 and 3), whereas, patients’ weight had a significant reduction in three study groups (*p* < 0.001 for three-group) (Table 2). However, the mean patients’ weight change in the video and face-to-face educational groups decreased more than the control group in a three-month period (mean changes= -2.37 Kg, -2.52 Kg, and − 1.31 Kg, respectively) (Table 2). The waterfall plots show the percentage of weight changes compared with baseline in each patient (Fig. 3). The plots show that weight was reduced compared to baseline in 98.3% (121) of participants in the video tele-education group and 95.8% (116) of participants in the in-person group, and the efficacy of both methods was the same.

### Subgroup analysis

Subgroup analysis by sex variable in the video tele-education group is shown in the supplementary Table S1 (Additional file 1). There was no significant difference between male and female and the efficacy of tele-education was almost the same in both groups.

### Patients’ satisfaction with the educational programs

The mean level of Patients’ satisfaction (scale from 0 to 10) with the in-person and video telecare educational programs were 5.91 ± 1.7 and 7.13 ± 1.80, respectively (*p* = 0.001).

## Discussion

We showed that both educational programs (face-to-face and video telecare educations) had relatively similar positive effects on the dietary regimen compliance in patients with T2DM. In both educational groups, significant reductions were observed in patients’ weight, TC, TG, V-LDL, and HbA1c, but reductions in FBS, LDL, and HDL were not significant. Dyson et al. [24] showed that a six-month educational program in newly diagnosed patients with T2DM resulted in significant reductions in TC, LDL, and HbA1c parameters. In a study conducted by Khan et al. [27], a three-month video telecare educational program improved patients’ self-care behaviors such as diet and medication compliance, knowledge about diabetes, exercise, and HbA1c’s control. Some studies similarly showed that video tele-education improved the HbA1c level in patients with diabetes [25, 26]. The results of this study were consistent with those of the above-mentioned studies suggesting that the video tele-educational program could improve dietary regimen compliance in patients with T2DM.

The results of the present study showed that face-to-face and video tele-educational programs had equal effects on dietary regimen compliance in patients with T2DM in a three-month period. This is consistent with the results of the studies by Baraz et al. (2010) and Hemmati et al. (2015), that compared face-to-face and video self-care education methods in patients undergoing hemodialysis and observed that they had equal effects [28, 29].

The link between patients’ weight and T2DM is very strong. hence, weight loss is widely recommended as key to management of T2DM [30]. Our study showed that Video telecare education was as effective as in-person education in controlling patients’ weight.

In the present study, the educational programs were delivered in both Farsi and Arabic languages. The multiplicity of different languages ​​in some areas makes the face-to-face education method much more difficult and the training may be missed. Prohibiting video contents can greatly address this challenge as well. Despite the advantages of in-person education, most of these methods are usually very brief and often does not offer adequate basic knowledge to patients [31]. Therefore, video telecare education has a supportive role in addition to other educational methods. Previous findings have shown that the use of educational technologies can improve self-care, as well as improve the relationship between healthcare professionals and patients [32]. This study also showed that the patients’ satisfaction in the video tele-education group was significantly higher than that in the in-person education group. Khan [27] also reported that multimedia-aided education was more accepted by both diabetic patients and healthcare staff. Marini [33] also reported that patients with venous thromboembolism were more satisfied with the video educational program. Clemensen et al. [23], assessed the home self-treatment of diabetic foot ulcers through video tele-education, and reported that all patients were satisfied with this type of education.

As an advantage of video telecare education, it can help educator specialists improve the basic knowledge and understanding of patients along with routine and face-to-face education. It is believed that the use of multimedia such as video increases motivation in patients to participate in decision-making, improves their knowledge of therapeutic objectives and reduces healthcare staff’s workload. Moreover, the use of such educational methods prior to other educational programs can motivate patients to learn more about diabetes’ self-care and improve their involvement in other educational programs [27].

The World Health Organization encourages use of telehealth, especially in low- and middle-income countries, as a new way of tackling serious health challenges [34]. In Iran, a low nurse-to-patient ratio (1.3 nurses per 1000 population), high nurses’ workload and lack of educator nurses which can bring patients’ education with challenges [35, 36]. Video tele-education have advantages, for example they are not restricted by availability of the nurse, and can be reviewed by patients whenever they prefer. Therefore, video telecare education can be used as an available, effective, ability to watch multiple times, simple, and low-cost method for improving patients’ self-care and quality of life. Patients’ perceptions of tele-care education also include a sense of focus, dominance, comfort, independence, and patient-centeredness, as well as less stress during training [37, 38].

### Limitations

The single-center, short follow-up period and lack of control of researchers on patients’ following up at home were some limitations of this study.

### Implications for clinical telehealth providers

Healthcare centers for patients with T2DM aim to improve the use of suitable educational methods for improving patient self-care behaviors and compliance with the therapeutic process. The improvement of self-care behaviors enhances the quality of life of patients and reduces diabetes educator nurses’ workloads in clinical settings. Video telecare education method can to standardize what patients learns, to adapt an easy to understand language, to make it culturally relevant, to be able to repeat it many times, to give them access to a link with the video at home so family members can learn with their loved ones.

## Conclusions

This study indicated that video telecare education is just as effective as in-person educational method, and was an effective method for providing education along with face-to-face nursing care which can positively influence the quality of telehealth care. In this investigation, video and face-to-face educations had equal effects on dietary regimen compliance in a three-month period in patients with T2DM. Therefore, it is recommended to use video telecare education in combination with or as an alternative to in-person education methods. Given the growing number of patients with T2DM, language-ethnicity diversities in some regions, high-cost of face-to-face education, lack of appropriate healthcare centers to educate patients in cities and villages, and insufficient number of diabetes education specialists in some regions, the use of video tele-education can largely compensate for traditional educational programs. Moreover, due to technological advances, video telecare education is a simple and a low-cost method, which is accessible at any time and place.

## Supplementary Information

**Additional file 1.**

## Data Availability

The datasets used and analysed during the current study are available from the corresponding author [MM] on reasonable request.

## Abbreviations

T2DM: Type 2 diabetes mellitus
TC: Total Cholesterol
FBS: Fasting Blood Sugar
HbA1c: Glycated Hemoglobin A1c
HDL: High-Density Lipoprotein Cholesterol
LDL: Low-Density Lipoprotein Cholesterol
V-LDL: Very Low-Density Lipoprotein Cholesterol
TG: Triglyceride
