# Supplementary material for: A randomized trial of comparing video telecare education vs. in-person education on dietary regimen compliance in patients with type 2 diabetes mellitus: a support for clinical telehealth Providers
Source: BMC Endocr Disord. 2022 May 2;22:116. doi: 10.1186/s12902-022-01032-4 (PMC9063130; doi:10.1186/s12902-022-01032-4)
Supplement: Supplementary file 1 — Additional file 1. [file 12902_2022_1032_MOESM1_ESM.docx]

**Table S1.** Subgroup analysis by sex variable in the video tele-education group

| **Parameters** | **Male (n=50)** | **Female (n=73)** | **P-value** |
| --- | --- | --- | --- |
| **TG**  Pretest  Posttest | 174.49±75.89  170.73±61.38 | 200.39±79.49  182.31±70.20 | 0.002*  0.064 |
| **HDL**  Pretest  Posttest | 42.98±7.60  43.43±7.37 | 43.46±7.13  44.05±6.54 | 0.026*  0.061 |
| **LDL**  Pretest  Posttest | 110.46±29.31  108.14±31.81 | 114.93±29.40  110.98±32.35 | 0.074  0.357 |
| **V-LDL**  Pretest  Posttest | 37.82±16.94  34.41±15.06 | 37.37±16.99  32.14±14.20 | 0.881  0.235 |
| **Total cholesterol**  Pretest  Posttest | 187.11±38.47  185.65±40.75 | 197.70±38.44  190.20±41.59 | 0.007*  0.198 |
| **FBS**  Pretest  Posttest | 169.40±72.27  159.95±59.75 | 160.89±79.56  162.36±72.23 | 0.084  0.372 |
| **HbA1c%**  Pretest  Posttest | 7.80±1.36  7.42±1.41 | 7.77±2.10  7.01±1.93 | 0.142  < 0.001* |
| **Weight (Kilograms)**  Pretest  Posttest | 91.89±16.09  89.84±15.76 | 92.37±15.02  90.27±15.10 | 0.623  0.726 |
| - SD: Standard deviation, TG: Triglyceride mg/dL, HDL: High-Density Lipoprotein Cholesterol mg/dL, LDL: Low-Density Lipoprotein Cholesterol mg/dL, V-LDL: Very Low-Density Lipoprotein Cholesterol mg/dL, FBS: Fasting blood Sugar mg/dL, HbA1c: Glycated Hemoglobin A1c% - Mann–Whitney U test was used. - * Statistically significant with a p value < 0.05. | | | |
